# Supercritical CO_2_ Extraction of Organic Solvents from Flunisolide and Fluticasone Propionate

**DOI:** 10.3390/pharmaceutics13050612

**Published:** 2021-04-23

**Authors:** Lucia Baldino, Mariarosa Scognamiglio, Ernesto Reverchon

**Affiliations:** Department of Industrial Engineering, University of Salerno, Via Giovanni Paolo II, 132, 84084 Fisciano, SA, Italy; mrscogna@unisa.it (M.S.); ereverchon@unisa.it (E.R.)

**Keywords:** supercritical CO_2_ extraction, flunisolide, fluticasone propionate, organic solvent extraction, active pharmaceutical ingredients

## Abstract

In this work, Class 2 and Class 3 solvents contained in two corticosteroids, flunisolide (Fluni) and fluticasone propionate (Fluti), were reduced to a few ppm by supercritical CO_2_ extraction. The process was carried out at pressures from 80 to 200 bar, temperatures of 40 °C and 80 °C, and at a fixed CO_2_ flow rate of 0.7 kg/h. The results demonstrated that CO_2_ density is the key parameter influencing the extraction kinetics and the solvent final residue. In particular, in the range investigated, optimal pressure and temperature conditions for the extraction of residual organic solvents were found working at 200 bar and 40 °C, which corresponds to a CO_2_ density of 0.840 g/cm^3^. Operating in this way, total organic solvent residues were reduced from 13,671 ppm and 326 ppm to 12 ppm and 10 ppm for Fluni and Fluti, respectively.

## 1. Introduction

Organic solvents are classified according to their dangerousness into three groups, in which Class 3 solvents are the lowest risk category [1,2]. The pharmaceutical industry is among the largest users of organic solvents, since, to produce active pharmaceutical ingredients (APIs), several processing steps are performed [3]. Therefore, to increase the safety of APIs, the reduction of these toxic organic solvents below the maximum residue limit is strongly recommended [3]. However, due to the physical and chemical barriers opposed by the solid structure of APIs, this operation is particularly difficult to carry out; moreover, high temperatures, required for solvent evaporation, should be not used, so as to avoid degradation of the active molecules [3,4,5,6].

Some drying methods have been proposed to solve this problem, such as fluidized bed drying, moving bed drying, spray drying, and static bed drying [3,4,5,6], but they present several limitations due to the cost, the chemical stability of the materials in contact with the organic solvents, and the scarce reproducibility of the purification performance.

An alternative solution to this issue could be the use of supercritical CO_2_ (SC-CO_2_). SC-CO_2_ is particularly appealing since is low-cost, inert, and non-toxic. It has been successfully adopted for the production of micro- and nanoparticles [7,8,9] and porous 3-D devices [10,11,12], for the extraction of active compounds from various vegetable matrices [13,14,15], and for cleaning [16]. Moreover, CO_2_ density—and its solvent power—can be modulated by the changing operative pressure and temperature [17,18,19], controlling, in this way, the selectivity and the kinetics of the process [20,21,22]. Finally, CO_2_ shows a large chemical affinity with almost all the organic solvents at mild process conditions, as a rule, at 40 °C and pressures near to 100 bar [23,24,25]. Once extracted from a solid matrix, organic solvents can be completely separated from CO_2_ by a simple depressurization step; this is a key characteristic of processing delicate active molecules.

Therefore, the scope of this work is to reduce/eliminate the Class 2 and Class 3 solvents contained in two corticosteroids (namely, flunisolide and fluticasone propionate) using supercritical CO_2_ extraction. The organic solvent residues are measured by gas chromatography using a flame ionization detector. Moreover, physicochemical characterizations of these APIs is also carried out to identify potential modifications after processing.

## 2. Materials and Methods

CO_2_ (99.9% purity) was purchased from Morlando Group SRL (Sant’Antimo (NA), Italy).

**Figure 1:**
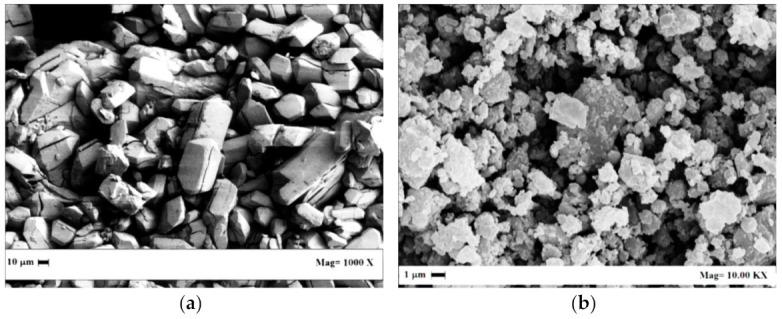
SEM images of (**a**) Fluni and (**b**) Fluti, as received.

Flunisolide (Fluni) and fluticasone propionate (Fluti) raw powders were kindly supplied by Genetic SpA (Fisciano (SA), Italy). SEM images of these APIs are reported in Figure 1a,b; they showed an irregular morphology at the micrometric scale. Organic solvent residues were identified and measured using a gas chromatograph (GC) interfaced with a flame ionization detector (FID); these data are reported in Table 1. All solvent residues belong to Class 3 solvents (limit 5000 ppm) except dichloromethane, which is a Class 2 solvent, and its recommended limit is below 600 ppm [1]. The sum of Class 3 solvent residues in Fluni was larger than the general limit of 5000 ppm recommended for this category of solvents [1]; this limit was not respected even considering only ethyl acetate content (9244 ppm). In the case of Fluti, only acetone residue was detected at 326 ppm; its processing was useful to demonstrate the possibility to largely overcome the limits set by the other processing alternatives.

Supercritical CO_2_ extraction experiments were performed using a laboratory apparatus equipped with a 0.2 dm^3^ internal volume stainless steel extractor. Four grams of API were mixed with 3 mm glass beads to minimize the possibility of caking and channeling phenomena. A stainless steel separator was located downstream of the extractor to collect the organic solvents. A piston pump (Gilson, mod. 305, Middleton, WI, USA) with a 25 SC pump head pumped liquid CO_2_ at the desired flow rate. CO_2_ was then heated to the extraction temperature using electrical thin bands; a thermocouple connected to a PID controller (Watlow, mod. 305, Corsico (MI), Italy) was used for the temperature measurement. A test gauge manometer (OMET, mod. MP1, Lecco, Italy) measured the pressure inside the extractor; this parameter was regulated by a micrometering valve (Hoke, mod. 1335G4Y, Spartanburg, SC, USA). CO_2_ flow rate was monitored by a rotameter (ASA, mod. d6, Sesto San Giovanni (MI), Italy) located after the separator. At regular time intervals, a small quantity of the solid material was sampled from the extractor after a slow depressurization of the system for chemical analysis. At the end of the process, the plant was slowly depressurized up to the atmospheric pressure. All experiments were performed in duplicate.

A headspace (HS) sampler (mod. 7694E, Hewlett Packard, Palo Alto, CA, USA) coupled with a gas chromatograph (GC) interfaced with a flame ionization detector (GC FID, mod. 6890 GC SYSTEM, Hewlett Packard, Palo Alto, CA, USA) was used to perform the solvent residue analysis. Solvents were separated using two fused-silica capillary columns connected in series by press-fit; the first column (mod. Carbomax EASYSEP, Stepbios, Bologna, Italy) was connected to the detector, 30 m length, 0.53 mm internal diameter, 1 µm film thickness, and the second one (mod. Cp Sil 5CB CHROMPACK, Stepbios, Italy) was connected to the injector, 25 m length, 0.53 mm internal diameter, 5 µm film thickness. HS conditions were as follows: oven temperature at 95 °C and manifold temperature at 105 °C; incubation time of 30 min. GC conditions were those described in [1]: oven temperature was set at 45 °C for 8 min; then, from 45 °C to 150 °C at 7 °C/min; from 150 °C to 210 °C at 38 °C/min; and, finally, at 210 °C for 6 min. Injector temperature was set at 100 °C, and detector temperature at 215 °C; carrier gas flux was 5 mL/min, using a split of 4:1 (about 20 mL/min); hydrogen flux was 30 mL/min, air flux 400 mL/min, and auxiliary flux 25 mL/min. Calibration curves were obtained for all solvents contained in the investigated APIs; the equations are as follows: y = 1.2438∙x for acetone, y = 0.8206∙x for ethyl acetate, y = 4.4263∙x for isopropyl ether, and y = 0.3609∙x + 1∙10^–14^ for dichloromethane. Analyses were performed in triplicate and an overall error of 2–3% was calculated, likely due to GC FID analysis and calibration curves.

A field emission scanning electron microscope (FE-SEM, mod. LEO 1525, Carl Zeiss SMT AG, Oberkochen, Germany) was used for the observation of API morphology. A small quantity of each API was dispersed on an aluminum stub (Agar Scientific, Stansted, UK), after which, it was coated with gold using a sputter coater (mod. 108 A, Agar Scientific, Stansted, UK) and, after that, observed by FE-SEM.

X-ray diffraction (XRD) was performed using a Bruker D8 X-ray diffractometer with CuKα radiation. Analyses were carried out using a 5 to 35 (2ϑ) scan with a 0.03 step.

Differential scanning calorimetric measurements were carried out using a Mettler Toledo DSC (TC11, Columbus, OH, USA) in a temperature range from 25 to 350 °C (15 °C/min heating rate) using nitrogen as an inert gas at a flow rate of 2 dm^3^/min.

## 3. Results and Discussion

The prerequisite for performing a successful extraction by SC-CO_2_ is that the compounds of interest to be extracted are largely soluble in SC-CO_2_. According to the scientific literature, all solvents considered in this work (i.e., the compounds of interest to be extracted in this case) are soluble in SC-CO_2_ at mild pressure (around 100 bar) and temperature (around 40 °C) [25,26,27,28,29,30]. APIs instead have to show either zero or very reduced solubility in SC-CO_2_ to avoid their co-extraction during processing. Velaga et al. [31] demonstrated that flunisolide has scarce solubility in SC-CO_2_ and, therefore, can be crystallized using the solution-enhanced dispersion by supercritical fluids (SEDS) technique. Vatanara et al. [32] studied the equilibrium solubilities of three glucocorticoid drugs at temperatures ranging from 65 to 85 °C and pressures from 213 to 385 bar in SC-CO_2_. However, in the case of fluticasone, in all these conditions, the solubility was too low for a correct determination. Therefore, data found in the literature demonstrate that the APIs tested in this work have scarce solubility in SC-CO_2_, as required. Starting from these considerations, specific ranges of extraction conditions were tested as follows: pressure from 80 to 200 bar, temperature of 40 °C and 80 °C, and CO_2_ flow rate was fixed at 0.7 kg/h. A reasonable hypothesis about the extraction process was, in this case, that an internal mass transfer resistance existed (i.e., organic solvents were entrapped inside the APIs’ solid structure), and CO_2_ flow rate should have a negligible influence on the extraction process.

In the following sections, the effects of pressure and temperature on the solvent extraction kinetics are discussed separately for each API.

### 3.1. Flunisolide

This corticosteroid contained acetone, ethyl acetate, isopropyl ether, and dichloromethane as solvent residues (see Table 1). The study was optimized considering acetone as the target solvent.

#### 3.1.1. Effect of Pressure

**Figure 2:**
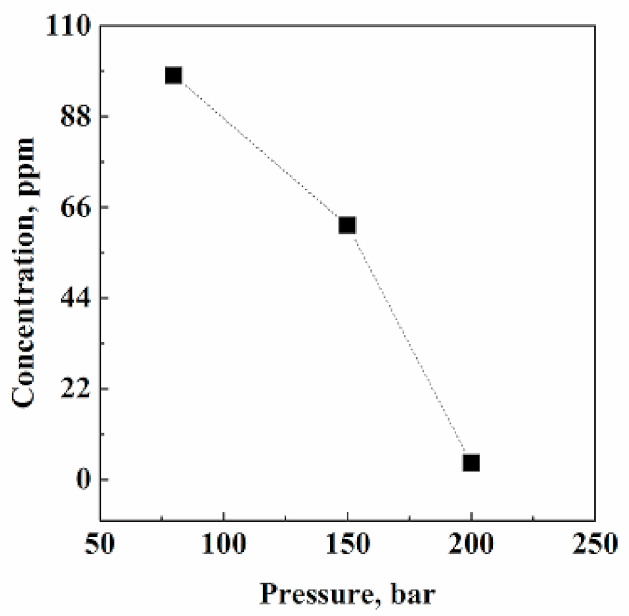
Acetone residual concentration in Fluni after SC-CO_2_ extraction performed at different operative pressures and at 40 °C and 0.7 kg/h CO_2_ flow rate.

Figure 2 reports the final acetone residues measured in Fluni after SC-CO_2_ extraction performed at 80, 150, and 200 bar, and at 40 °C. Extraction efficiency largely increased with pressure, since, from 80 bar to 200 bar, the percentage of acetone removed from Fluni passed from 11% to about 96%. An explanation of this trend can be related to CO_2_ solvent power. Specifically, working at 40 °C and increasing pressure from 80 to 200 bar, CO_2_ density changes from 0.281 to 0.840 g/cm^3^, respectively. This strong increase in CO_2_ density largely increased the capability of CO_2_ to penetrate the API solid matrix and to solubilize the organic solvent, achieving a final acetone residual content of 4 ppm at 200 bar.

Looking only at the solubility indications, liquid solvents’ massive elimination should be expected operating at lower pressures. However, considering that these residues are somewhat linked to the solid structure, the addition of a relatively strong internal mass transfer resistance largely justify the observed results.

#### 3.1.2. Effect of Temperature

**Figure 3:**
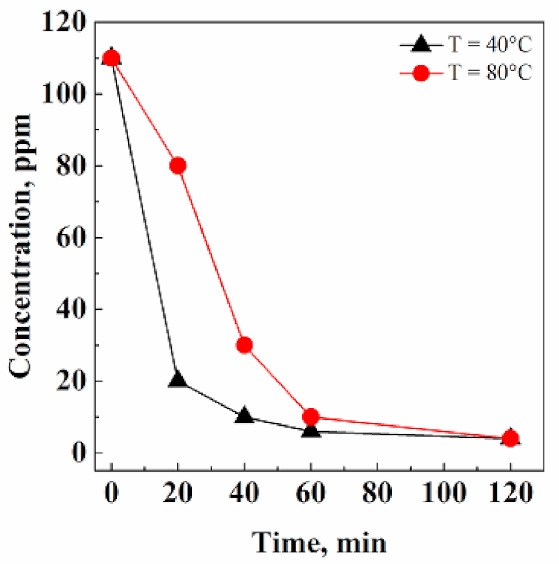
Effect of temperature on acetone residual concentration in Fluni, investigated at 200 bar and 0.7 kg/h CO_2_ flow rate.

The possible influence of temperature on the solvent extraction performance was investigated at 40 °C and 80 °C, maintaining the operative pressure constant at 200 bar and the CO_2_ flow rate at 0.7 kg/h. As shown in Figure 3, acetone extraction kinetics were faster operating at 40 °C. This behavior was due to the different CO_2_ densities at 40 °C and 80 °C operating at 200 bar. In particular, it was 0.840 g/cm^3^ at 40 °C, and 0.594 g/cm^3^ at 80 °C. A larger CO_2_ density determines a larger CO_2_ solvent power [33] and, therefore, an improved extraction capability from the solid matrix. Moreover, when working at a mild temperature (i.e., 40 °C), potential thermal stresses on the API are avoided.

#### 3.1.3. Extraction Kinetics

**Figure 4:**
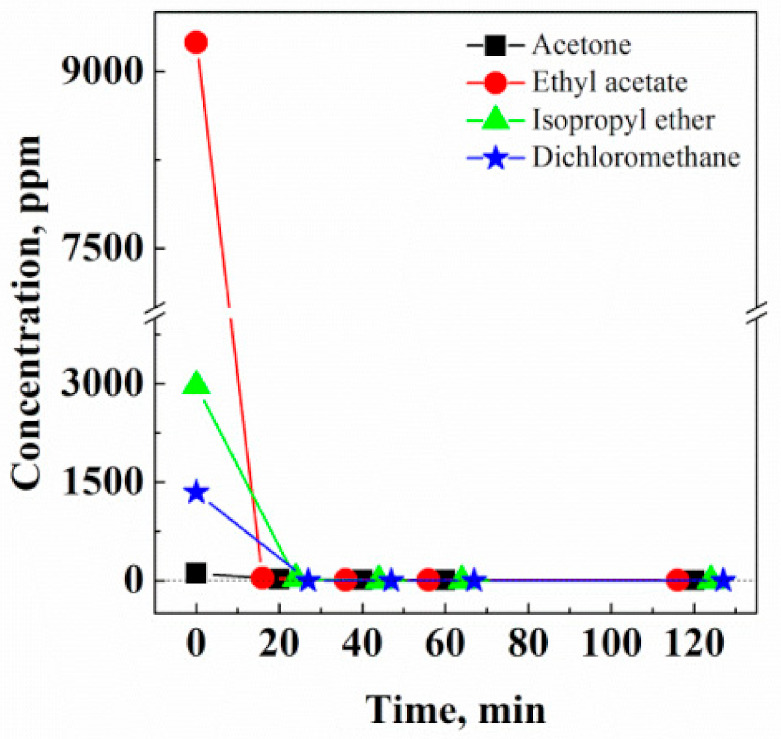
Solvent extraction kinetics from Fluni, working at 200 bar, 40 °C, and 0.7 kg/h CO_2_ flow rate.

Once the optimal extraction conditions operating at 200 bar and 40 °C were determined using a 0.7 kg/h CO_2_ flow rate, the extraction kinetics for all the solvents present in Fluni were studied under these conditions. The results are shown in Figure 4; Table 2 reports the solvent residue values measured in Fluni at the end of the extraction process.

Figure 4 demonstrates that the residues of the four solvents detected in Fluni rapidly reached near zero ppm. In particular, after 40 min of supercritical extraction, solvent residue values were 4 ppm and 8 ppm for acetone and ethyl acetate, respectively, whereas isopropyl ether and dichloromethane residual contents were 0 ppm. The sum of all solvent residues after the treatment was 12 ppm.

### 3.2. Fluticasone Propionate

The series of extraction tests described previously was also performed on Fluti, with the difference that, in this API, only acetone was present; its initial content, measured by GC FID, was 326 ppm. Additionally, in this case, the impact of pressure and temperature on the solvent extraction performance was investigated at a fixed CO_2_ flow rate of 0.7 kg/h.

#### 3.2.1. Effect of Pressure

**Figure 5:**
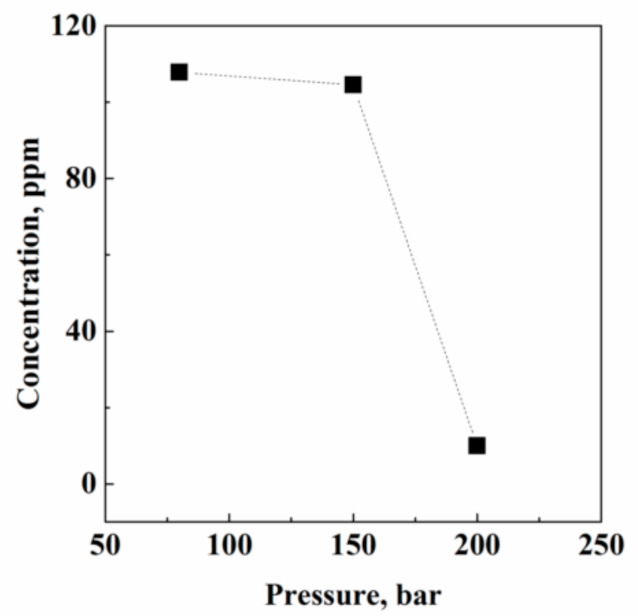
Acetone residual concentration in Fluti after SC-CO_2_ extraction performed at different operative pressures and at 40 °C and 0.7 kg/h CO_2_ flow rate.

Additionally, for this API, extraction efficiency largely increased with pressure. The diagram reported in Figure 5, referring to acetone content, clearly shows this tendency. In this case, a final acetone residual content of 10 ppm was reached at 200 bar (Table 2).

#### 3.2.2. Effect of Temperature and Extraction Kinetics

**Figure 6:**
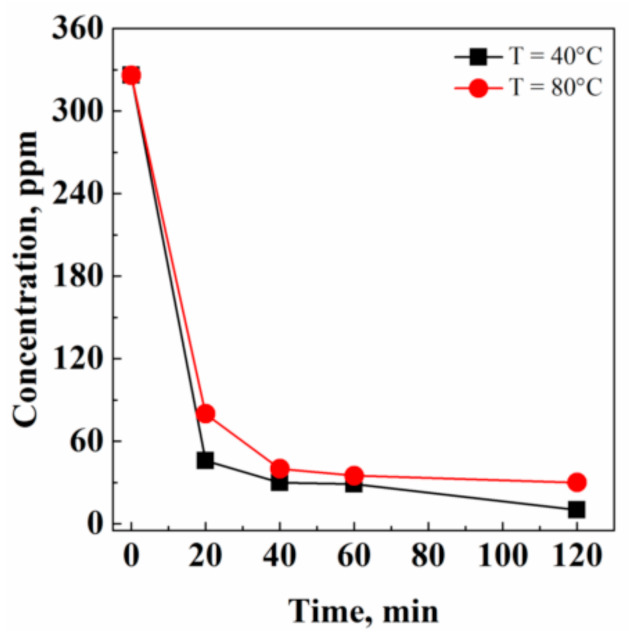
Effect of temperature on acetone residual concentration in Fluti investigated at 200 bar and 0.7 kg/h CO_2_ flow rate.

As for Fluni, the same trend as for the extraction kinetics with temperature was observed for this API. Specifically, beside a faster acetone extraction, a lower solvent residual value was also measured at the end of the process performed at 40 °C and 200 bar, i.e., 10 ppm instead of 30 ppm was measured, as reported in Figure 6.

### 3.3. Other Characterizations

A series of SEM, XRD, and DSC analyses was performed on the native and SC-CO_2_-treated APIs to determine if modifications occurred due to the solvent extraction treatment.

**Figure 7:**
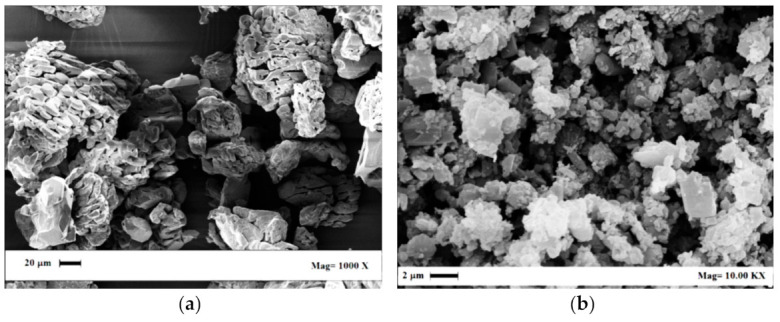
SEM images of the solid materials after the supercritical extraction process: (**a**) Fluni and (**b**) Fluti.

Comparing the SEM images reported in Figure 7a,b with the ones of Figure 1a,b, Fluni resulted in slight modifications after processing, since a porosity appeared on the treated material (Figure 7a); whereas Fluti preserved the starting morphology and mean size (Figure 7b).

**Figure 8:**
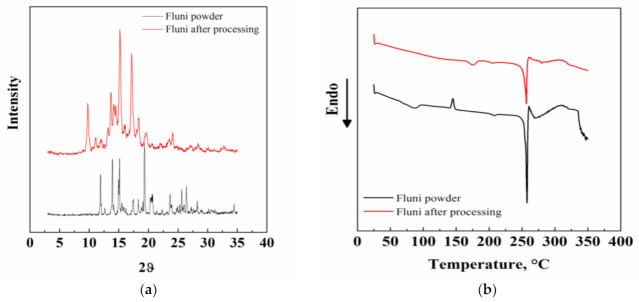

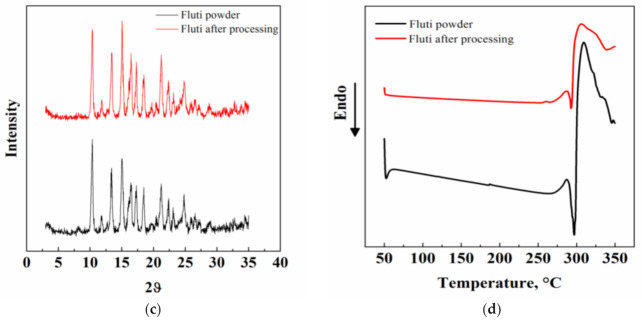
Fluni XRD patterns before and after the supercritical extraction in (**a**); Fluni DSC thermograms before and after the supercritical extraction in (**b**); Fluti XRD patterns before and after the supercritical extraction in (**c**); Fluti DSC thermograms before and after the supercritical extraction in (**d**).

Some modifications were also detected in the XRD and DSC patterns of Fluni, as reported in Figure 8a,b. In particular, this material evidenced a solid–solid transition from a polymorphic form III before processing to a polymorphic form II in the supercritical treated API (Figure 8a). This kind of transition has also been observed previously in this API and in other materials processed by SC-CO_2_ [31]. Figure 8b reports the Fluni melting temperature at 257.6 °C [31].

No relevant modifications were observed instead in the XRD and DSC patterns of Fluti before and after SC-CO_2_ extraction (Figure 8c,d), i.e., this API remained in the same crystalline form (Figure 8c) [32] with a melting temperature at 300 °C (Figure 8d) [34].

## 4. Conclusions and Perspectives

A successful reduction/elimination of organic solvent residues present in the selected APIs was obtained by SC-CO_2_ extraction. In particular, isopropyl ether and dichloromethane were completely eliminated from Fluni; whereas, acetone residual content was 4 ppm and 10 ppm in Fluni and Fluti, respectively. These results were obtained in a very short extraction time, as a rule, around 40 min, up to a maximum of 120 min. In the range investigated, optimal pressure and temperature conditions for the extraction of residual organic solvents were found working at 200 bar and 40 °C, corresponding to a CO_2_ density of 0.840 g/cm^3^. At these operative conditions, the overall final content of organic solvents was reduced to 12 ppm and 10 ppm for Fluni and Fluti, respectively.

In perspective, the negligible effect of the CO_2_ flow rate on the supercritical extraction of organic solvents from APIs hypothesized in this work should be verified, performing experiments at the optimal pressure and temperature, and by changing CO_2_ flow rate. Moreover, a study on the correlation between the chemical affinity among organic solvent/solid matrix/SC-CO_2_ and their extraction kinetics should be performed to identify general indications to also be applied to other solid pharmaceutical systems.

## Figures and Tables

**Table 1:** Solvent residues contained in Fluni and Fluti, as received.

API	Acetone, ppm (Class 3)	Ethyl Acetate ^1^, ppm (Class 3)	Isopropyl Ether, ppm (Class 3)	Dichloromethane ^1^, ppm (Class 2)	Initial Overall Organic Solvent Residues, ppm
Fluni	110	9244	2970	1347	13,671
Fluti	326	-	-	-	326

^1^ Single solvent content above the regulatory limit [1].

**Table 2:** Solvent residues contained in Fluni and Fluti at the end of the supercritical extraction process.

API	Acetone, ppm (Class 3)	Ethyl Acetate, ppm (Class 3)	Isopropyl Ether, ppm (Class 3)	Dichloromethane, ppm (Class 2)	Final Overall Organic Solvent Residues, ppm
Fluni	4	8	0	0	12
Fluti	10	-	-	-	10

## Data Availability

Not applicable.
